# Incorporation of Different Metal Ion for Tuning Color and Enhancing Antioxidant Activity of Curcumin/Palygorskite Hybrid Materials

**DOI:** 10.3389/fchem.2021.760941

**Published:** 2021-12-13

**Authors:** Shue Li, Bin Mu, Penji Yan, Yuru Kang, Qin Wang, Aiqin Wang

**Affiliations:** ^1^ Key Laboratory of Clay Mineral Applied Research of Gansu Province, Center of Eco-Material and Green Chemistry, Lanzhou Institute of Chemical Physics, Chinese Academy of Sciences, Lanzhou, China; ^2^ Center of Materials Science and Optoelectronics Engineering, University of Chinese Academy of Sciences, Beijing, China; ^3^ Key Laboratory of Hexi Corridor Resources Utilization of Gansu Universities, College of Chemistry and Chemical Engineering, Hexi University, Zhangye, China

**Keywords:** curcumin, palygorskite, metal ions, color tuning, antioxidant activity, stability

## Abstract

Curcumin is one of the dietary dyes extracted from turmeric and used for prevention and treatment of various illnesses. However, the low bioavailability and poor stability of curcumin limits its relevant applications. Therefore, different metal ions including Cu^2+^, Zn^2+^, Mg^2+^, Al^3+^, or Fe^3+^ were incorporated to tune the color, enhance the environmental stability and antioxidant activity of curcumin in the presence of palygorskite in this study. The as-prepared samples were studied using X-ray diffraction, Fourier transform infrared spectroscopy, X-ray photoelectron spectroscopy, Zeta potential, and transmission electron microscopy. In addition, the density functional theory calculation was also performed to explore the possible interaction among metal ions, curcumin and palygorskite. It was found that the color changing and stability enhancing were ascribed to the curcumin-metal ions coordination as well as chemical interactions between curcumin-metal complex and palygorskite. Moreover, the as-prepared composites showed more excellent color, thermal stability, antioxidant activity, and fluorescence properties than that of the curcumin/palygorskite composites due to the presence of metal ions. The finding of this investigation may contribute to developing the multifunctional composites with different colors and good antioxidant activity for relevant applications based on curcumin and palygorskite.

## Introduction

It has long been known that many over-the-counter natural products can be used to treat and prevent a variety of chronic diseases. Among them, some of dietary polyphenols including curcumin (Cur), anthocyanin and resveratrol have attracted considerable attention because of their useful therapeutics (Jin 2018). In particular, Cur, a kind of naturally occurring yellow polyphenols, was extracted from the rhizomes of Curcuma longa or turmeric and used for centuries in varieties pharmaceutical applications towards various diseases due to the general biological and pharmaceutical activity (Barik et al., 2007; Kumar et al., 2020b). As a result, the green and low-toxic index natural product was widely used as traditional medicine, food coloring and flavoring agent, and a cosmetic agent for skin care for its antioxidant activities, anti-thrombotic and other health benefits (Pourreza et al., 2018; Lin et al., 2020). From the structure of Cur molecule (1,7-bis-(4-hydroxy-3-methoxyphenyl)-1,6-heptadiene-3,5-dione), it has a lot of available metal binding sites. Cur chelate with metal ions through the central β-diketone functionality of molecular structure to form the more stable complexes and scavenge the reactive oxygen species (Zhao et al., 2010). Beyond that, one hydroxyl group attached on each of the two benzene rings plays a major role in antioxidant activity of Cur (Priyadarsini et al., 2003). At present, there are many reports that the metal ions complexes of Cur are very useful for curing various diseases due to their higher anti-oxygen activity, better antimicrobial property and aqueous stability, as well as antitumor and anticancer effect (Barik et al., 2007; Refat, 2013; Zhang et al., 2016; Mary et al., 2018; Wang et al., 2020).

**GRAPHICAL ABSTRACT:**
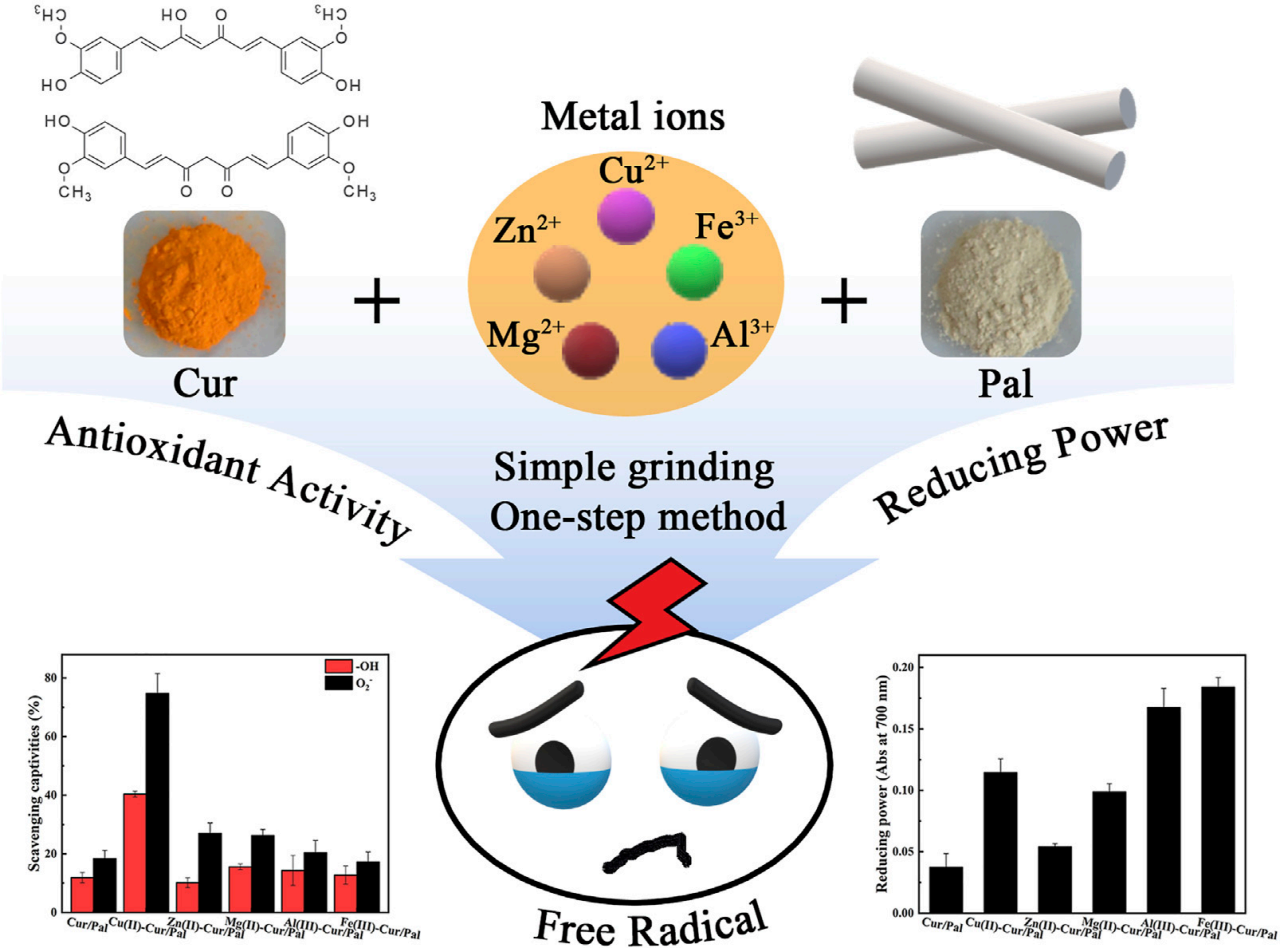


However, the poor stability of Cur reduced the acceptance of consumers, which restricted the application of Cur as a functional ingredient in food (Hu et al., 2020). As a result, a number of strategies including encapsulation into liposomes (Cheng et al., 2017), the loading of nanoparticles (Arab et al., 2021; Yuan et al., 2021), chemical modifications by polymer conjugates (Li et al., 2020b; Chen et al., 2020), plasmonic nanogel (Howaili et al., 2020), and complexation with proteins (Mohammadian et al., 2019; Hu et al., 2020) have been applied to stabilize Cur and expand its relevant applications. But beyond all that, clay minerals deserve more attention because of their safety, low-cost and excellent performances. To the authors’ knowledge, there are still very few studies on Cur and clay minerals in the last few years. Previously, Cur-Au hybrid nanoparticles loaded halloysite nanotubes were developed by *in-situ* preparation method and then coated with bio-adhesive chitosan, making them exhibit the NIR responsive and pH-responsive properties (Rao et al., 2018). Some research had focused on the development of montmorillonite systems, with or without modification, as a kind of Cur carrier in order to achieve Cur stabilization and improve its availability (Madusanka et al., 2015; Gonalves et al., 2017).

It is well-known that palygorskite (Pal), a nanorod-like hydrated magnesium-rich aluminosilicate clay mineral, is renowned for its ability to stable natural indigo dye (Xu et al., 2017). What’s more, Pal possessed regular nanochannel structure has unique crystal morphology, large active surface groups and specific surface area, resulting in the good adsorption efficiency. As early as 1966, van Olphen confirmed, as Dogenech-Carbo et al. later demonstrated, that Pal was more beneficial than other clay minerals in stabilizing the natural indigo molecules (Van Olphen, 1966; Domenech-Carbo et al., 2013). To date, in order to stabilize natural pigments, such as betanin, flavylium cations and anthocyanin, and expand their application fields, Pal-based hybrid pigments have been developed due to the excellent performances, such as superior stability, fluorescence and acid/base reversible allochroic behavior (Silva et al., 2018; Li et al., 2019a; Li et al., 2020a). In this context, Pal is an attractive supporting matrix that can be employed as an inorganic carrier to stabilize Cur molecules.

In this paper, Cur/Pal and a series of metal ion-Cur/Pal hybrid materials with different yellow hues were prepared. The main objective of this research is to explore the effect of the different metal ion on the color, thermal stability, antioxidant activity, and fluorescence properties of Cur/Pal. Based on their excellent properties, the synthesized the multifunctional composites with different colors and good antioxidant activity had the good application prospects.

## Experimental

### Materials and Methods

Palygorskite (Pal) was supplied by Guanshan Mine, Anhui Province, China. Curcumin (Cur) was purchased from the Afaisha (China) Chemical Co., Ltd., Shanghai, China. Other chemicals were of analytical grade from commercial sources.

### Synthesis of Metal Ions-Cur/Pal Hybrid Materials

Metal ions-Cur/Pal hybrid materials were synthesized employing the simple one-step method by mixing Cur with copper acetate monohydrate, zinc acetate dihydrate, magnesium acetate tetrahydrate, aluminium chloride hexahydrate, and ferric chloride at a molar ratio of 1:2, 1:1, 1:1, 1:2, and 1:2, respectively. Briefly, 0.1359 g of copper acetate monohydrate, 0.2989 g of zinc acetate dihydrate, 0.2908 g of magnesium acetate tetrahydrate, 0.1637 g of aluminium chloride hexahydrate or 0.1834 g of ferric chloride was added into 0.5 g of Cur dissolved in 2 ml ethanol with 4 ml distilled water with a pH of 4.0 adjusted by HCl aqueous solutions (except for copper acetate monohydrate), respectively. And then, 1 g of Pal was added to the above mixture and ground under dark conditions for 30 min to obtain a series of samples. In order to remove the excess Cur and metal ions, the mixture was washed with 100 ml deionized water and anhydrous ethanol for three times, respectively, after vacuum drying at 60°C for 2 h. It was worth noting that the samples of each washing were centrifuged at 4,000 rpm for 10 min to achieve solid-liquid separation. Finally, the final products were obtained after being placed overnight in a vacuum drying oven at 60°C. The samples were labeled as Cu(II)-Cur/Pal, Zn(II)-Cur/Pal, Mg(II)-Cur/Pal, Al(III)-Cur/Pal, and Fe(III)-Cur/Pal, respectively. The sample without metal salts involved in the preparation process was labeled as Cur/Pal.

### Characterization

The Fourier transform infrared (FTIR) spectra, transmitting electron microscopy (TEM), powder X-ray diffraction (XRD) analysis, Zeta potentials, Field emission transmission electron microscopy (TEM), and X-ray photoelectron spectroscopy (XPS) of the samples were obtained according to our previously reported methods (Li et al., 2020a). The elemental analysis of all samples was determined by Elementar analyzers varioEL cube (Elementar Analysen systeme GmbH, Germany). Thermogravimetric analysis (TGA) was conducted on a STA8000 simultaneous thermal analyzer (PerkinElmer, United States) at a heating rate of 10°C min^−1^ under a N_2_ atmosphere, and the initial mass of all samples was about 0.005 g. The color parameters of all as-prepared samples were evaluated using a Color-Eye automatic differential colorimeter (X-Rite, Ci 7800, Pantone Inc, United States) on the basis of the CIE 1976 *L* ^*^ *a* ^*^ *b* ^*^ colorimetric method, in which *L* ^*^ is the color lightness (from 0 (black) to 100 (white)), negative/positive *a* ^*^ is the red/green axis, and negative/positive *b* ^*^ is the yellow/blue axis. Chromaticity (*C* ^*^) and hue angle (*h°*) are calculated by Eqs 1, 2:

$$C^{*}={\left\{{\left(a^{*}\right)}^{2}+{\left(b^{*}\right)}^{2}\right\}}^{1/2}$$ (1)

$$h^{{}^{\circ}}=\arctan\left(b^{*}/a^{*}\right)$$ (2)

The color difference in the CIE *L* ^*^ *a* ^*^ *b* ^*^ space was evaluated using *ΔE* ^*^ calculated according to Eq.3:

$$\Delta E^{*}={\left\{{\left(\Delta L^{*}\right)}^{2}+{\left(\Delta a^{*}\right)}^{2}+{\left(\Delta b^{*}\right)}^{2}\right\}}^{1/2}$$ (3)

Furthermore, the reflectance and corresponding UV-vis spectra were obtained from X-Rite, Ci 7800.

### Computational Details and Models

In order to cognize the loading mechanism, the DFT and TD-DFT calculations were performed based on the Gaussian 09 (Frisch et al., 2009) program and carried out using M062X (D3) (Zhao and Truhlar 2008; Grimme et al., 2010) function combined with the 6-31G(d) basis set for Si, O, Mg, Al, and H. The Stuttgart/Dresden effective core pseudopotentials (SDD) basis set was used for Cu, Zn, and Fe. Based upon the optimized structures, the UV-visible spectra properties of the Cur molecules and metal-Cur complexes, metal-Cur-Pal complexes were calculated adopting the time-dependent DFT (TD-DFT) method with the B3LYP(D3BJ)/6-31G(d) (Becke 1993; Grimme 2006; Schwabe and Grimme 2007) basis set in dimethyl sulfoxide (DMSO) media with the Solvation Model Density (SMD) (Marenich et al., 2009) implicit solvation model. The electrostatic potential (ESP) was generated on vdW surface depended on Multiwfn 3.8 (Lu and Chen 2012) and was visually presented using Visual Molecular Dynamics (VMD) software (Humphrey et al., 1996). We have used Gauss View program (Dennington et al., 2009) to generate ball and stick geometries of optimized structures.

### Thermal Stability of Metal Ions-Cur/Pal Hybrid Materials

The thermal stabilities based on colour change of Cur/Pal and series of metal ions-Cur/Pal hybrid materials were tested by placing the samples in an oven at 120, 150, 180, and 210°C for 30 min, respectively.

### Fluorescence Properties of Metal Ions-Cur/Pal Hybrid Materials

The fluorescence spectra of DMSO suspension of Cur, Cur/Pal and series of metal ions-Cur/Pal hybrid materials was conducted using a HORIBA Fluorolog-3 spectrofluorometer (HORIBA Instruments Inc, United States), and recorded by monitoring fluorescence excitation at 435 nm.

### Free Radical Scavenging Experiments

The superoxide free radical scavenging ability of the as-prepared samples was determined by the pyrogallol autoxidation method described previously with slight modifications (Li, 2012). 200 μL DMSO containing sample was mixed with 2,700 μL of Tris-HCl buffer (0.05 M, pH 8.2) and then heated in a water bath at 37°C for 4min after shaken vigorously for several seconds. Subsequently, 100 μL of pyrogallol solution (60 mM in 1 mM HCl, 37°C) was added to the above mixture, immediately vigorously shaken, and then send to measure its absorbance at 325 nm every 30 s for 5 min using UV-vis spectrometer (UV-1900, Shimadzu, Japan). The superoxide free radical scavenging ability was calculated using the following Eq. 4:

$$Scavenging\;ability\left(\%\right)=\left\{\left(\Delta A_{0}-\;\Delta A_{S}+\Delta A_{S0}\right)/\Delta A_{0}\right\}\times 100\%\;$$ (4)

where, *ΔA*_*0*_ is the increase of absorbance at 325 nm in 5 min of the mixture without the sample and *ΔA*_*S*_ is that of the mixture with the sample, respectively. *ΔA*_*S0*_ is the absorbance at 325 nm of sample without pyrogallol solution. The assay was repeated for three times.

The ability of scavenging the hydroxyl radicals for all samples were tested the absorbance at 510 nm, according to the previous report (Wei et al., 2021). At first, 1.8 M ferrous sulfate solution, 1.8 M hydrogen peroxide solution and 1.8 M salicylic acid-ethanol solution were prepared in advance. After that, solution *A*_*0*_ was obtained by adding salicylic acid-ethanol solution (2 ml), ferrous sulfate solution (2 ml) and hydrogen peroxide solution (2 ml) into DMSO (2 ml). In the case of the sample, 2 ml of salicylic acid-ethanol solution, ferrous sulfate solution, hydrogen peroxide solution and sample solution were mixed to obtain solution *As*. 2 ml of salicylic acid-ethanol solution, ferrous sulfate solution and sample solution was mixed to prepare solution *A*_*S0*_. Finally, the absorbance of three solutions at 510 nm was determined after being incubated at 37°C for 30 min. The hydroxyl free radical scavenging rate can be calculatedby following formula:

$$\;Scavenging\;ability\left(\%\right)=\left\{\left(A_{0}-\;A_{S}+A_{S0}\right)/A_{0}\right\}\times 100\%$$ (5)

### Reducing Power

Reducing power of the as-prepared samples was determined according to the previous method (Gulcin et al., 2004; Hu et al., 2020). Each sample DMSO solution (1 ml) was blended with PBS (2.5 ml, 0.2 M, pH 6.6) and K_3_Fe(CN)_6_ solution (2.5 ml,1%, w/v), and then incubated in water bath at 50°C for 20 min. After that, trichloroacetic acid (2.5 ml,10%, w/v) was added and centrifuged at 4,000 rpm for 10 min. Supernatant (2.5 ml) was blended with 2.5 ml of deionized water and 0.5 ml FeCl_3_ solution (0.1%,w/v), and stored at room temperature for 10 min. The absorbance at 700 nm of each sample was then documented. It should be noted that higher absorbance of the reaction mixture demonstrated greater reductive potential.

## Results and Discussion

### Synthesis and Characterization of Metal Ions-Cur/Pal

**FIGURE 1:**
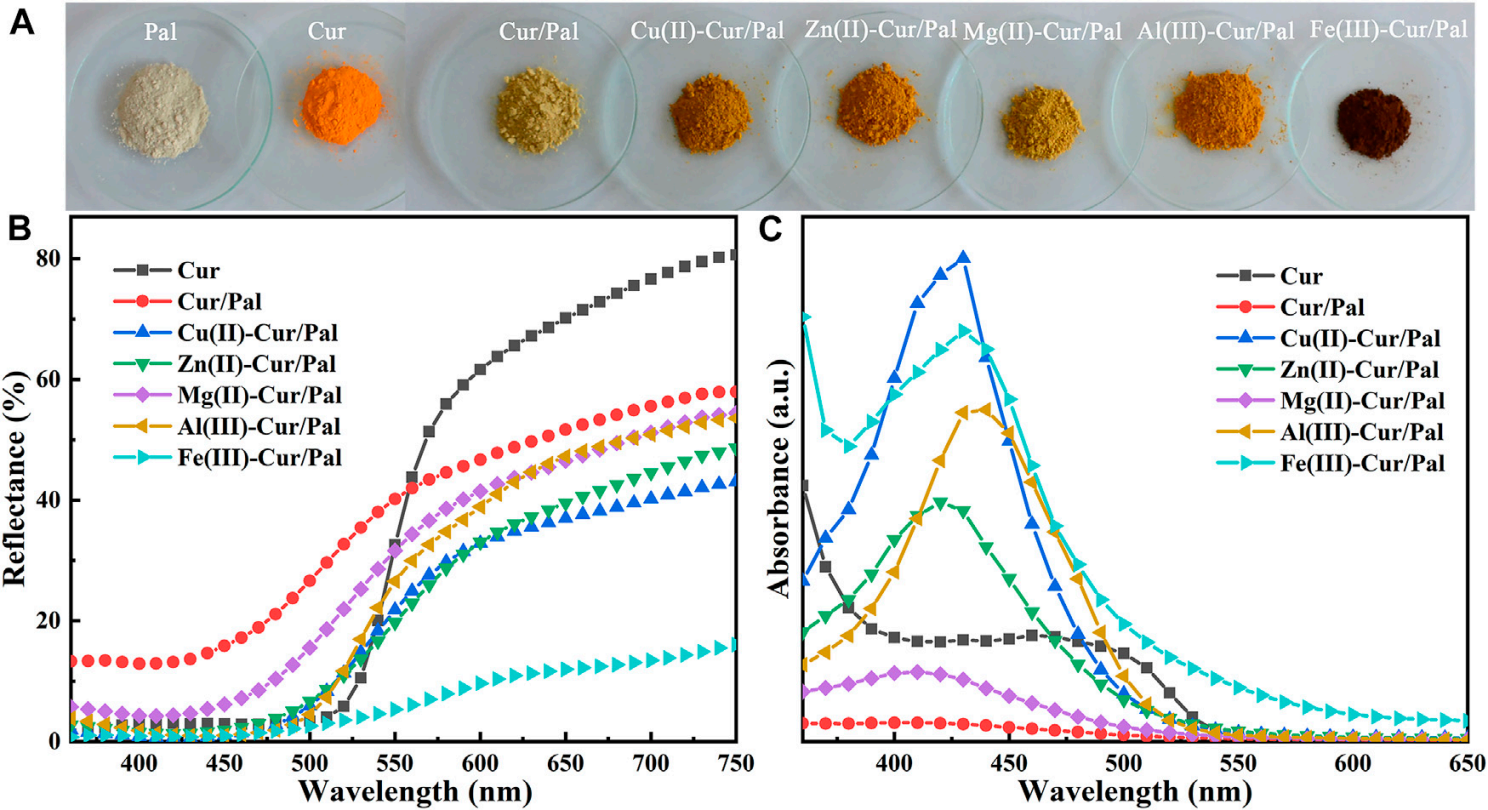
**(A)** Digital images, **(B)** UV-vis diffuse reflectance spectra and **(C)** UV-vis spectra of Cur, Cur/Pal and metal ions-Cur/Pal hybrid materials.

Digital images, UV-vis diffuse reflectance spectra and UV-vis spectra of Cur, Cur/Pal and metal ions-Cur/Pal hybrid materials are shown in Figure 1. Effects of Cu^2+^, Zn^2+^, Mg^2+^, Al^3+^, and Fe^3+^ on the color of Cur/Pal hybrid materials were investigated. It was obviously observed that Pal and Cur are off white and saffron yellow in color, respectively. Compared with pure Cur, the yellow color of the Cur/Pal hybrid materials became lighter obviously after introduction of Pal. However, the tone of the hybrid materials was significantly changed by adding metal ions (Figure 1A; Supplementary Figure S1). As shown in Supplementary Table S1, a* and b* values of Cur/Pal with different metal ions (except for Fe^3+^) increased obviously, which was suggested that the redness and yellowness of metal ions-Cur/Pal were enhanced compared with that of Cur/Pal. Among them, Al(III)-Cur/Pal presented the reddest and yellowest values, suggesting that Al^3+^ was more conducive to the color enhancement of Cur/Pal. In the same case, *L* ^*^ values representing brightness was reduced and the color of the obtained samples became darker than Cur/Pal. The above results indicated that the participation of metal ions might improve the loading capacity of Pal to Cur (Gutiérrez et al., 2017). In addition, the increase in the *C* ^*^ values and decrease in the *h* ^*°*^ values were also observed when Cu^2+^, Zn^2+^, Mg^2+^, Al^3+^, and Fe^3+^ was incorporated into Cur molecule and Pal, respectively. It indicated that the metal ions-Cur/Pal samples presented a more vivid color in yellow or orange zone (Li et al., 2019b). In addition, the reflectance spectra of hybrid materials prepared with different metal ions, Cur, and Pal were evaluated (Figure 1B), and the strong absorption mainly of all samples happened in wavelength of 400–550 nm of the visible region, including the blue and green light region (Wang et al., 2018). Hence, these samples presented their complementary color of yellow or orange-yellow. Moreover, a blue shift on the optical absorption bands was also observed for all metal ions-Cur/Pal hybrid materials compared with that of the pure Cur (Figures 1B,C).

**FIGURE 2:**
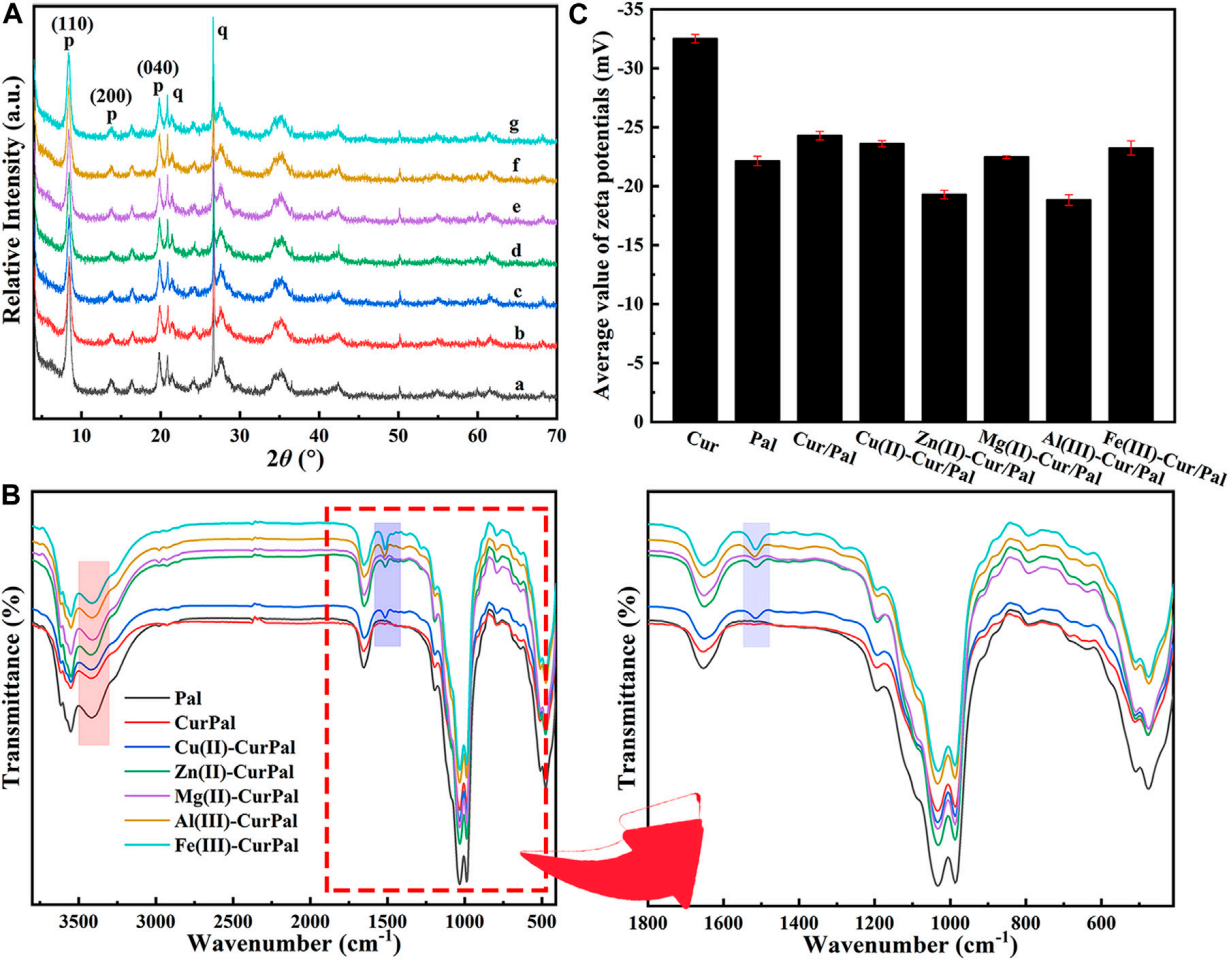
**(A)** XRD patterns, **(B)** FTIR spectra and local magnification, and **(C)** Zeta potential of Pal, Cur/Pal and metal ions-Cur/Pal hybrid materials.

Figure 2A is the XRD patterns of Pal, Cur/Pal and the corresponding metal ions-Cur/Pal hybrid materials. The characteristic diffraction peaks of Pal crystal have been determined from the diffractograms, which was consistent with those reported in the literatures (Li et al., 2019a; Lu et al., 2019). In addition, small amounts of quartz in Pal had been found. However, no characteristic peaks of Cur crystal phases were observed for the Cur/Pal and metal ions-Cur/Pal hybrid materials due to the transformation from crystalline to the X-ray amorphous nature of Cur (Kumar et al., 2020a). FTIR spectra of as-prepared Cur/Pal and metal ions-Cur/Pal hybrid materials were shown in Figure 2B. In the spectrum of Pal, the absorption peak observed in the wavelength between 3,430 cm^−1^and 3,400 cm^−1^ was due to the hydroxyl stretching vibration of coordinated water located at terminal positions of the octahedral sheets, physical-adsorbed water on Pal surface and zeolitic H_2_O inside Pal channels (Zhang et al., 2015a; Carazo et al., 2018; Li et al., 2019a). The OH bending vibration of the above water molecules appeared at ca. 1650 cm^−1^ (Miao et al., 2020). In detail, the hydroxyl stretching and bending vibrations were shifted from the values of 3,416 and 1653 cm^−1^ for Pal to 3,409 and 1650 cm^−1^ for Cur/Pal, 3,419 and 1650 cm^−1^ for Cu(II)-Cur/Pal, 3,422 and 1650 cm^−1^ for Zn(II)-Cur/Pal, 3,410 and 1651 cm^−1^ for Mg(II)-Cur/Pal, 3,414 and 1650 cm^−1^ for Al(III)-Cur/Pal, 3,413 and 1651 cm^−1^ for Fe(III)-Cur/Pal, respectively. Significantly, both bands were shifted in the Cur/Pal and metal ions-Cur/Pal hybrid materials because of the H-bonding of carbonyl group of Cur with complexion water existed in the channels of Pal. In addition to the characteristic peaks of Pal, the new characteristic band associated with C=C stretching vibration of benzene rings of Cur was also observed at 1515 cm^−1^ in the spectrum of metal ions-Cur/Pal hybrid materials (Madusanka et al., 2015; Zhou and Tang 2016). However, the bands of Cur/Pal were identical to that of Pal except in their intensities because of the low concentration of Cur in Pal, which resulted in no clearly visible bands being observed (Ma et al., 2017).

Cur had a Zeta potential value of −32.50 mV, which was due to the negative charged phenol groups (Figure 2C) (Liao et al., 2018). The Zeta potential was −24.30 mV, −23.6 mV, −19.3 mV, −22.47 mV, −18.83 mV, and −23.23 mV for Cur/Pal, Cu(II)-Cur/Pal, Zn(II)-Cur/Pal, Mg(II)-Cur/Pal, Al(III)-Cur/Pal, and Fe(III)-Cur/Pal, respectively, after incorporation of Pal and different metal ions. Compared with Pal (−22.13 mV), the Zeta potential values of as-prepared samples remained negative. Remarkably, the slight increase in the Zeta potential values of Zn(II)-Cur/Pal and Al(III)-Cur/Pal indicated the formation of electrostatic interaction. In the cases of Cur/Pal, Cu(II)-Cur/Pal, Mg(II)-Cur/Pal, and Fe(III)-Cur/Pal, hydrogen bonding interaction between compositions should predominate in such systems.

**FIGURE 3:**
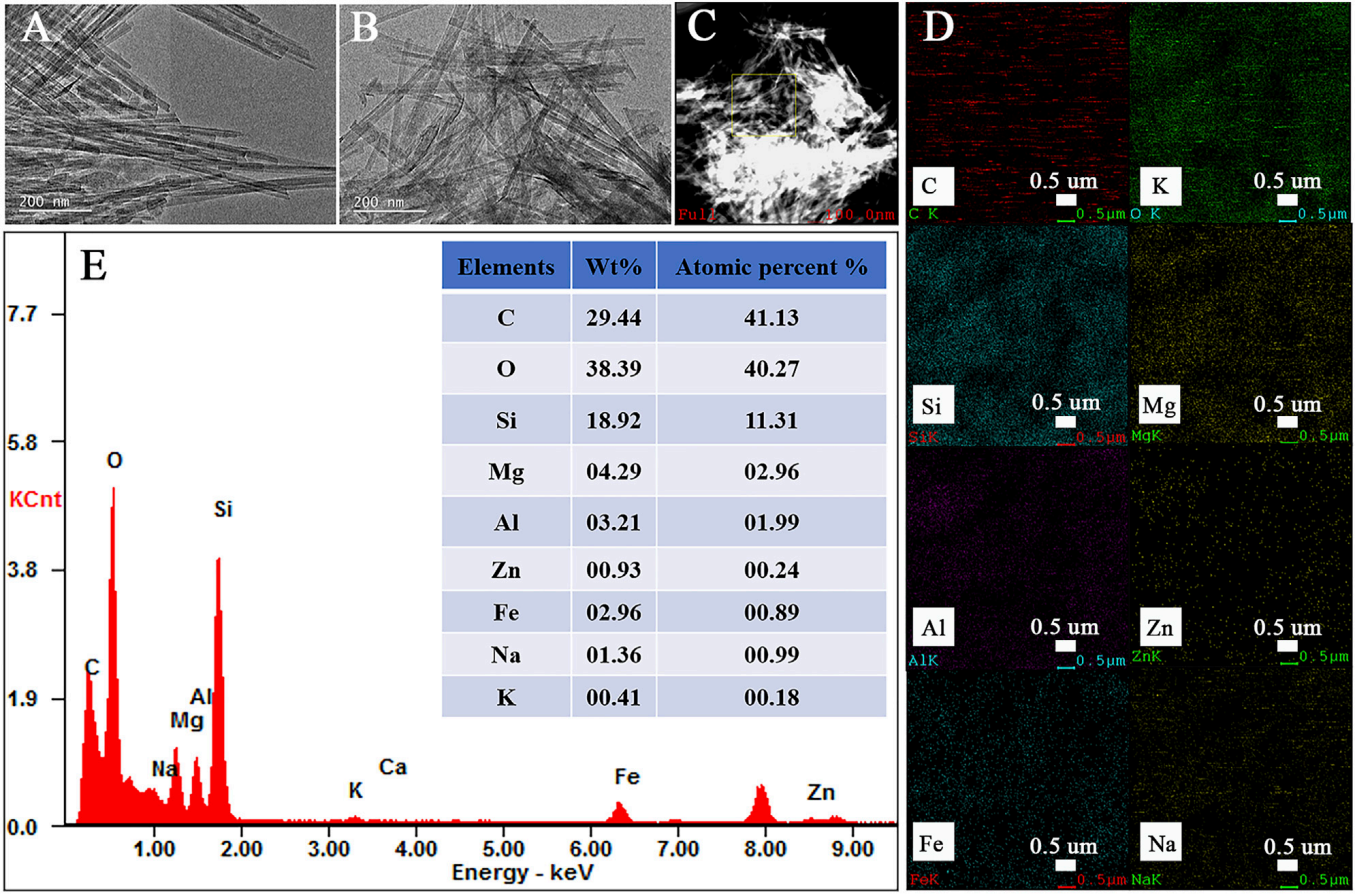
TEM of Pal **(A)**, the elemental mappings **(B–D)** of various elements and EDS spectrum of Zn(II)-Cur/Pal hybrid material (**E**).

The major elements within the Zn(II)-Cur/Pal hybrid material are qualitatively detected by EDS elemental analysis (Figure 3). As shown in Figures 3A,B, it was worth mentioning that the representative rod-like morphology of Pal was retained and the crystal bundles in the sample were better disaggregated by grinding. The elemental mappings and EDS spectrum are presented in Figures 3D,E, which confirmed that the distribution of C, O, Si, Mg, Al, Zn, Fe, Na, and K is highly uniform in Zn(II)-Cur/Pal samples, indicating that Zn(II)-Cur complex was formed on Pal matrix. In addition, the elemental analysis (C%) of the Cur/Pal and metal ions-Cur/Pal hybrid materials was listed in Supplementary Table S2, in which C% of Cur/Pal, Cu(II)-Cur/Pal, Zn(II)-Cur/Pal, Mg(II)-Cur/Pal, Al(III)-Cur/Pal, and Fe(III)-Cur/Pal hybrid materials was 1.26, 2.41, 2.07, 1.61, 2.33, 3.51%, respectively. It indicated that the content of Cur in as-prepared metal ions-Cur/Pal samples was higher than that of Cur/Pal.

**FIGURE 4:**
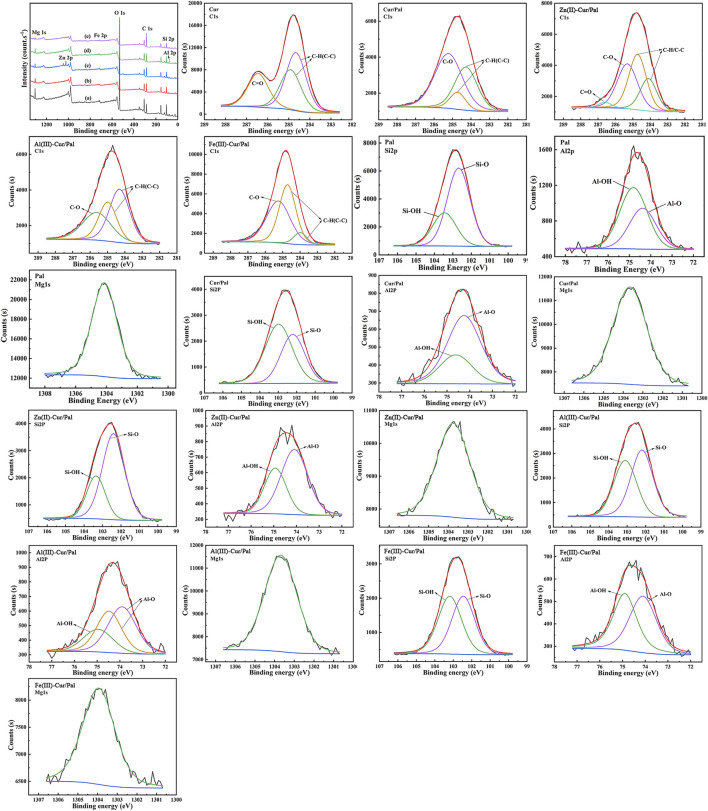
XPS survey of Pal **(a)**, Cur/Pal **(b)**, Zn(II)-Cur/Pal **(c)**, Al(III)-Cur/Pal **(d)**, and Fe(III)-Cur/Pal **(e)** and high-resolution XPS spectra of C1s, Si2p, Al2p, and Mg1s.

XPS analysis elucidate the chemical state of the surface C1s, Si2p, Al2p, Mg1s, Zn2p, Fe2p element content in the surface analyses of the Cur, Pal, Cur/Pal, Zn(II)-Cur/Pal, Al(III)-Cur/Pal, and Fe(III)-Cur/Pal hybrid materials. The wide range spectra of the Pal, Cur/Pal, Zn(II)-Cur/Pal, Al(III)-Cur/Pal, and Fe(III)-Cur/Pal are presented in Figure 4. As expected, the pristine Pal surface was characterized by the existence of Si, Al, Mg, Fe, and O, as well as some adventitious C (Supplementary Table S3). After treatment, the O1s, Si2p, Al2p, and Mg1s peaks were weaker compared with the spectrum of Pal, whereas the C1s peak was much stronger in as-prepared samples. The high C content in the Cur/Pal, Zn(II)-Cur/Pal, Al(III)-Cur/Pal, and Fe(III)-Cur/Pal hybrid materials was owing to the presence of Cur. In addition, Zn(II)-Cur/Pal exhibited one new low intensity peaks at 1022 eV compared with Cur/Pal, which was in keeping with Zn2p valence band region. Moreover, there are differences in C1s spectra in Cur, Pal and as-prepared samples as shown in Figure 4, Supplementary Figure S2 and Supplementary Table S4. In the case of Cur, the Cls region could be fitted into three peaks located at around 284.66, 284.91 and 286.45 eV, which were ascribed to C-C and C-H linkages, C=O bonds, respectively (Mandal et al., 2017; Moradi et al., 2020). Compared with Cur and Pal, the new peak of C1s at a binding energy of 285.22 eV related to the C-O bond became remarkably visible while the peak corresponding to C=O bonds gradually disappeared in the XPS spectra of as-prepared samples. What’s more, the Si2p, Al2p, and Mg1s peaks of samples slightly shift to lower binding energy than that of Pal with an decrease in the intensity. The above results of XPS also proved the present of Cur on the surface of Pal by complexation with metal ions.

**FIGURE 5:**
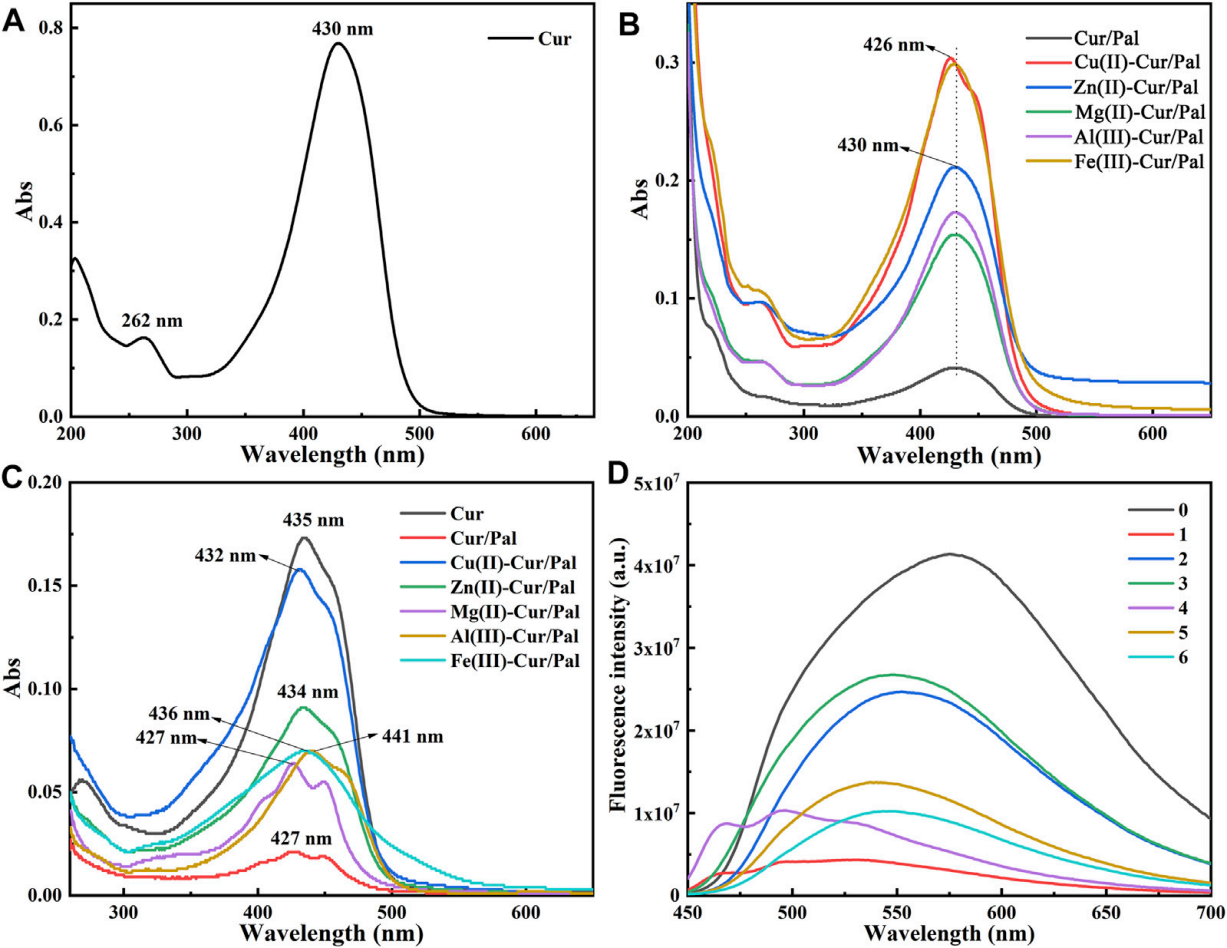
UV-spectra of Cur, Cur/Pal and metal ions-Cur/Pal hybrid materials dissolved in 80% ethanol/water solution **(A,B)** and in DMSO **(C)**. **(D)** Fluorescence emission spectra of the above samples [0: Cur; 1: Cur/Pal; 2: Cu(II)-Cur/Pal; 3: Zn(II)-Cur/Pal; 4: Mg(II)-Cur/Pal; 5: Al(III)-Cur/Pal; 6: Fe(III)-Cur/Pal].

The UV-Vis absorption spectra of Cur, Cur/Pal and metal ions-Cur/Pal hybrid materials in 80% water-ethanol are depicted in Figures 5A,B. The spectrum of Cur exhibited a weak absorption in the UV region attributing to the phenolicmoiety and the absorption maximum at 430 nm in the visible region due to the keto-enol moietyin a polar protic solvent (Beneduci et al., 2019). The addition of the metal cations of Zn^2+^, Mg^2+^, Al^3+^, or Fe^3+^ into Cur and Pal resulted in the marked difference in their intensity, though the peaks showed no obvious shift compared with that of Cur. Notably, the peak wavelength of Cu(II)-Cur/Pal was around 426 nm and had an evident shoulder at higher wavelength with respect to Cur, which was consistent with previous reports (Picciano and Vaden, 2013). As depicted in Figure 5C, their spectra exhibited absorption maxima bands at 435 nm for Cur, at 427 nm for Cur/Pal, at 432 nm for Cu(II)-Cur/Pal, at 434 nm for Zn(II)-Cur/Pal, at 427 nm for Mg(II)-Cur/Pal, at 441 nm for Al(III)-Cur/Pal, at 436 nm for Fe(III)-Cur/Pal, respectively. The bands existed at 400–470 nm region could be correspond to an n→π^*^ transitions type (Goswami et al., 2013; Refat 2013). The slight shift could be detected on the Cur/Pal and metal ions-Cur/Pal hybrid materials, indicating that the carbonyl group of Cur was involved in the reaction (Song et al., 2009; Refat 2013). It was vital that the spectra of Cur/Pal was similar to that of Mg(II)-Cur/Pal except for strength, suggesting that Cur might have formed a complex with OH_2_, which was coordinated with Mg^2+^ at the edges of the octahedral layers of Pal.

The fluorescence emission spectra of the above-mentioned samples are presented in Figure 5D. In the case of Cur, the peak at ∼575 nm was due to fluorescence emission. Upon the addition of various metal ions, it was interesting to note that the peak of the samples showed obvious blue shift compared with that of Cur. However, the fluorescence emission spectra of Cur/Pal and Mg(II)-Cur/Pal were similar (Figure 5D), that is, the shoulder appeared in the lower wavelength except for the blue shift. Therefore, it could be considered that the hypochromatic shifted emission of Cur/Pal and metal ions-Cur/Pal hybrid materials produced excitation in Cur monomers (Bhopate et al., 2015). What’s more, although internal quenching of Cur would occur in the presence of metal due to complexation, the fluorescence intensity of metal ions-Cur/Pal was still obviously enhanced compared with Cur/Pal because of the higher loading of Cur. Among them, the strongest fluorescence in Zn(II)-Cur/Pal sample were observed, followed by Cu(II)-Cur/Pal, Al(III)-Cur/Pal, Mg(II)-Cur/Pal, and Fe(III)-Cur/Pal in sequence.

### Structural Models of Cur/Pal and Metal Ions-Cur/Pal on DFT Calculation

As shown in Supplementary Figure S3, there are two possible structures of Cur, one is ketone structure named as Cur I, and the other is enol structure named as Cur II. The *ΔG* of Cur II in aqueous solution is 3.97 kcal/mol, which is lower than that of Cur I due to the formation of intramolecular hydrogen bonds in Cur II. Therefore, Cur II is more stable than Cur I. The ESP values showed that the minimum value was at the carbonyl group, indicating that the coordination with metal ions mainly occurred at the carbonyl group.

All the possible structural models of coordination structures of metal ions-Cur are presented in Supplementary Figure S4. Among them, M-CurI-1 represented the coordination of metal ions with two carbonyl groups of Cur I, M-CurI-2 referred to the coordination of metal ions with one carbonyl group of Cur I while the other carbonyl group was rotated to one side of the molecule through the σ bond. It was worth noting that M-Cur II-1 was converted to M-CurI-2 due to the metal coordination with the carbonyl group, breaking the intermolecular hydrogen bond force. Both M-Cur I-3/4 and M-Cur II-2 were considered to coordinate one metal ion with two Cur molecules. As shown in Supplementary Figure S5, the intramolecular hydrogen bond was broken after the metal coordinated with the carbonyl group of Cur II, leading to the enol structure being converted into ketone, and the converted carbonyl group was rotated to the other side of the molecule due to influencing factors such as sterichindance. Surprisingly, Supplementary Figure S6 suggested that the UV spectra of various structures were basically consistent with the experimental values. Therefore, it could be concluded thatthe coordination modes between metal ions and Cur was mainly M-Cur I-2, while that of Al ions was Al(III)-Cur I-1 mode. Furthermore, the possible adsorption of Cur by Pal was analyzed in Supplementary Figure S7. It found that carbonyl group of Cur II formed hydrogen bond with complexion water existed in the channels of Pal rather than coordinated with Mg^2+^ at the edges of the octahedral layers, resulting in destroying the intramolecular hydrogen bonds of Cur to form Cur I/Pal mode. In the cases of Al(III)-Cur I-1/Pal and Zn(II)-Cur I-2/Pal, both of them were coordinated with Al^3+^ and Zn^2+^ by Si-OH on Pal surface (Supplementary Figure S8). In conclusion, the coordination of metal ions with Cur and Si-OH of Pal as well as the hydrogen bond interaction with complexion water existed in the channels of Pal significantly improved the loading capacity of Pal for Cur.

### Thermal Stability of Metal Ions-Cur/Pal

**FIGURE 6:**
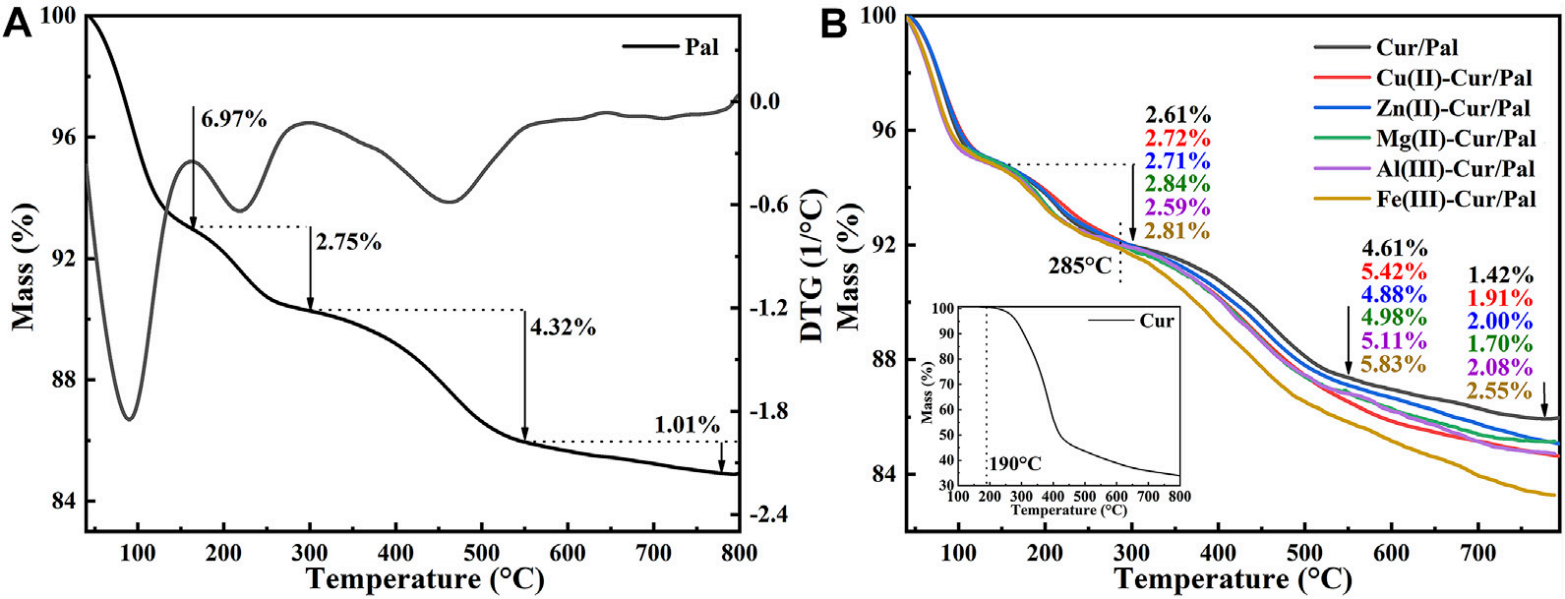
TGA curves of **(A)** Pal, Cur (the inset), **(B)** Cur/Pal and metal ions-Cur/Pal hybrid materials.

The thermal stability of hybrid materials is of great significance for practical application. The TGA curves of Pal, Cur/Pal and metal ions-Cur/Pal hybrid materials are displayed in Figure 6. In the case of Pal (Figure 6A), it presented four stages of mass loss, consistent with those previously reported (Zhang et al., 2015b). Briefly, the mass loss before 160°C was attributed to the loss of adsorbed water and loosely bound zeolitic H_2_O (6.97% weight loss). The small mass loss at 170°C–300°C, corresponding to the 2.75% weight loss, was associated with the mass loss of residual zeolitic H_2_O and the first fraction of structural OH_2_. The third step occurring at the range of 300°C–550°C was assigned to the remove of residual structural OH_2_, and the weight loss in this step was about 4.32%. The last 1.01% weight loss occurred at 550°C–800°C, corresponding the degradation of structural OH_2_ and hydroxyl groups. The decomposition of Cur began at about 190 °C, ascribing to the dehydroxylation of Cur molecules (Figure 6B) (Varaprasad et al., 2020). The total mass loss of pure Cur below 500°C reached 43.59%. The thermal decomposition of the prepared samples was the same as that of Pal, which was also divided into four steps. However, the mass losses were obviously different after the temperature reached about 285°C by comparison different metal ions-Cur/Pal samples, which was due to the decomposition of different amounts of Cur in samples. Obviously, it suggested the thermal stability of as-prepared metal ions-Cur/Pal samples was higher than that of pure Cur.

**FIGURE 7:**
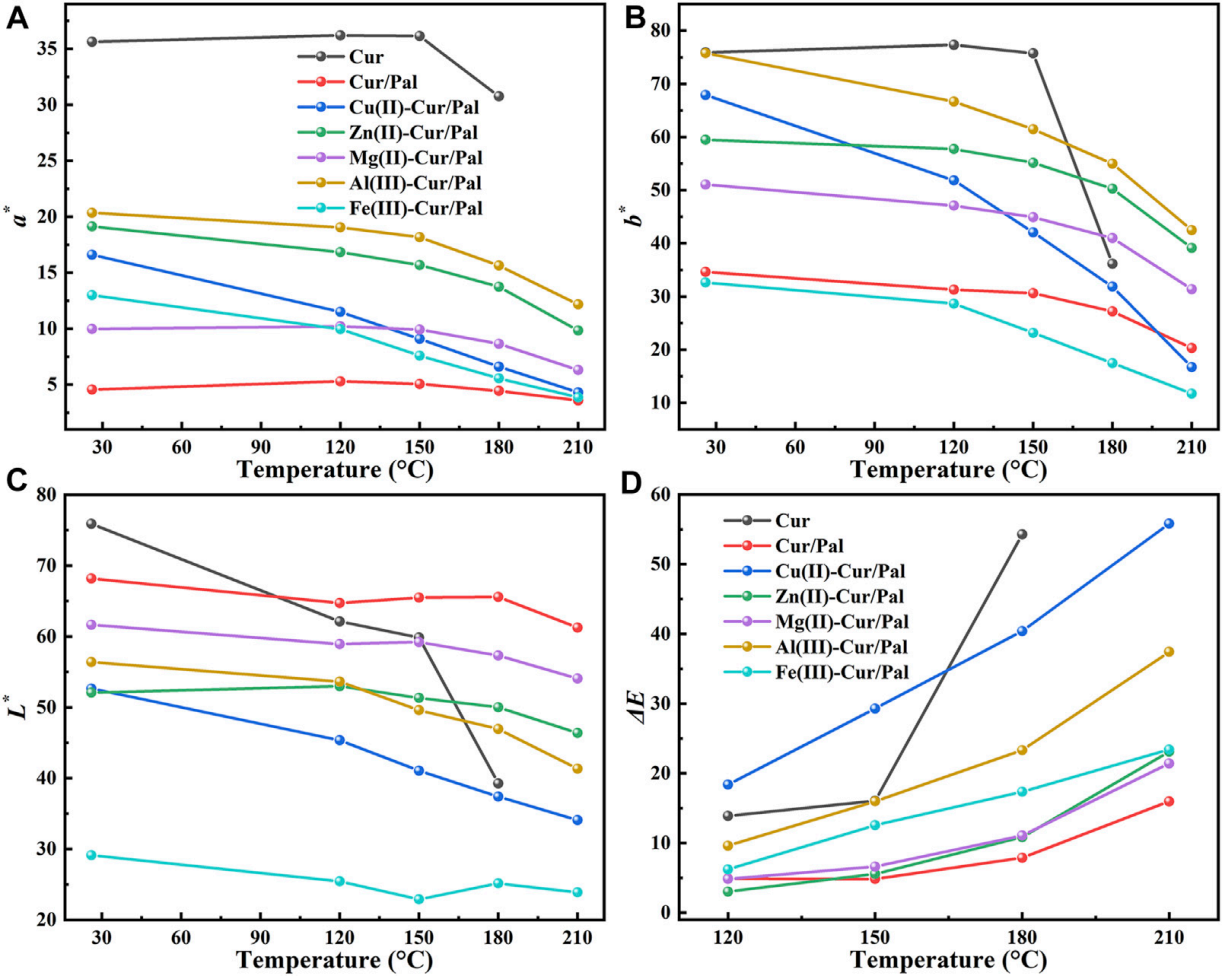
CIE **(A–C)** and ΔE* **(D)** values of Cur, Cur/Pal and metal ions-Cur/Pal hybrid materials after being heated at different temperatures.

The thermal stabilities of as-prepared samples were further estimated by heat treating in an oven at 120°C, 150°C, 180°C, and 210°C for 30 min, respectively. The differences in the CIE and color differences (*ΔE* ^*^) values of Cur, Cur/Pal and metal ions-Cur/Pal hybrid materials heated at different temperatures are presented in Figure 7. Obviously, the highest *L* ^*^, *a* ^*^ and *b* ^*^ values was obtained for the pure Cur, demonstrating the vivid color. There was no significant variation in *a* ^*^ and *b* ^*^ values after being heated to 150°C, but these values dropped sharply when Cur was heated at 180°C, and the degradation occurred as temperatures continued to rise. The *L* ^*^ value of Cur decreased sharply with the increase of temperature, indicating a gradual dimming of brightness (Figures 7A–C). Although the *ΔE* ^*^ value of Cur/Pal changed slightly at different temperatures, the *a* ^*^ and *b* ^*^ values of Cur/Pal were obviously lower than that of metal ions-Cur/Pal (Figure 7D). It might be attributed to the fact that metal ions contributed to enhancing the loading capacity of Cur on Pal, resulting in the initial huge of the samples was significantly superior to Cur/Pal. The above result was consistent with the thermogravimetric data. Furthermore, the prepared samples exhibited a much more limitedchanges in color, *L* ^*^, *a* ^*^, *b* ^*^, and *ΔE* above 150°C compared with pure Cur (Supplementary Figure S9), showing the optimal thermal stabilities.

### Antioxidant Activity of Metal Ions-Cur/Pal

**FIGURE 8:**
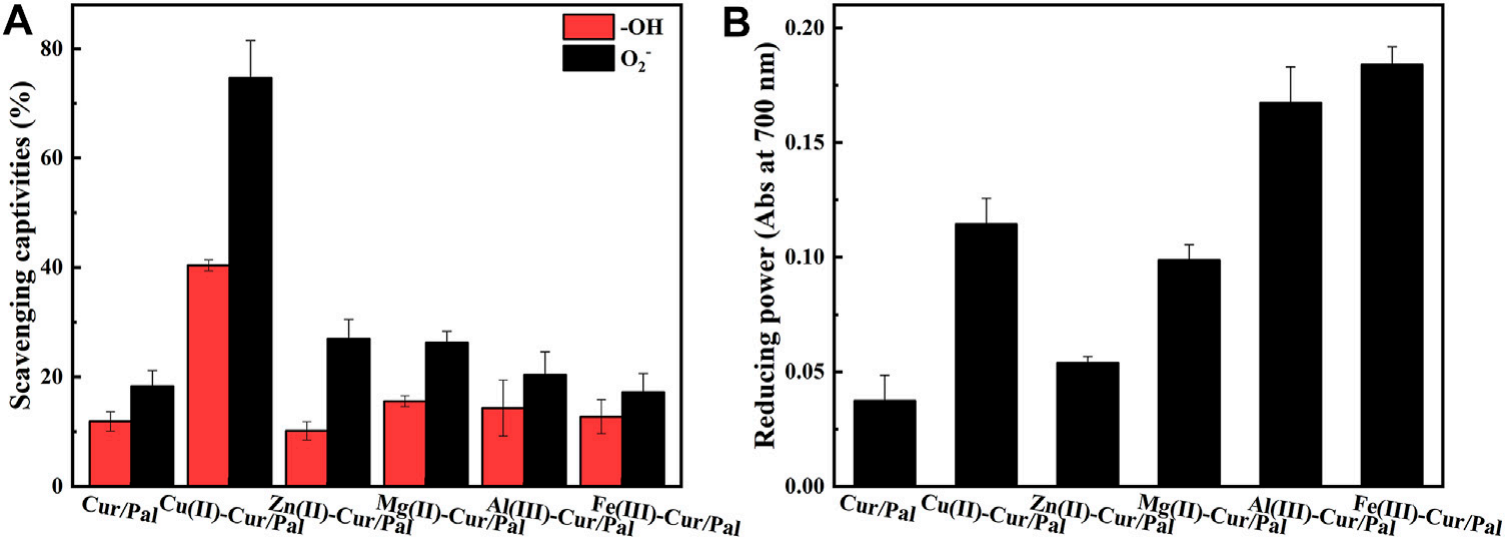
Antioxidant activity **(A)** and reducing power **(B)** of Cur/Pal and metal ions-Cur/Pal hybrid materials.

It has been established that Cur has excellent antioxidant properties and is a powerful scavenger of various reactive oxygen species due to the presence of the two phenol-OH group, the enol form of the diketone moiety, and the extended conjugated structure (Ak and Gulcin 2008; Shakeri et al., 2019). Figure 8 demonstrates antioxidant activity and reducing power of Cur/Pal and metal ions-Cur/Pal hybrid materials. It was obvious that the metal ions-Cur/Pal had increased free radical scavenging activity compared with Cur/Pal (Figure 8A). The significantly improved antioxidant activity of the metal ions-Cur/Pal complexes might be due to the acquisition of additional superoxide dismutating centers (De Souza and De Giovani, 2004; Shakeri et al., 2019). Among them, Cu(II)-Cur/Pal possessed the highest antioxidant activity, which could be ascribed to the weaker O-H bond exist in the case of Cu(II)-Cur, resulting in easier H-atom loss process in *ο*-methoxy phenolic group (Bagchi et al., 2015). In the rest metal ions-Cur/Pal samples, the existence of stronger O-H bond resulted in the radical scavenging capacity lesser than that of Cu(II)-Cur/Pal (Zhao et al., 2010; Bagchi et al., 2015). Cur with stable electron donor properties could neutralize free radicals by forming stable products, resulting in the termination of the radical chain reactions (Ak and Gulcin 2008). As presented in Figure 8B, the result of radical scavenging activity was in line with the reducing power. Of note, ferrous reducing power activities of metal ions-Cur/Pal were also much more effective and powerful reducing ability than the Cur/Pal.

## Conclusion

In conclusion, the Cur/Pal and metal ions-Cur/Pal complexes with different huge were designed and synthesized based on natural Cur dye and Pal by a simple method, in which natural Pal was served as effective carrier for loading the Cur-metal ions complex. The effect of different metal ions including Cu^2+^, Zn^2+^, Mg^2+^, Al^3+^, and Fe^3+^ on the huge, environmental stability, antioxidant activity, and fluorescence properties of Cur in the presence of Pal was evaluated. In the case of Cur/Pal, Cur molecule might have formed a complex with H_2_O, which was coordinated with Mg^2+^ along the edges of the octahedral layers of Pal. Compared with Cur/Pal composites, the as-prepared composites showed more excellent properties due to the presence of metal ions. The results indicated that the color change and stability enhancement were attributed to the coordination of metal ions with Cur and Si-OH of Pal as well as the hydrogen bond interaction with complexion water existed in the channels of Pal. Therefore, the as-prepared multifunctional composites with different colors and good stability present the potential applications in the relevant fields based on Cur and Pal.

## Data Availability

The raw data supporting the conclusion of this article will be made available by the authors, without undue reservation.
