# Supplementary material for: Incorporation of Different Metal Ion for Tuning Color and Enhancing Antioxidant Activity of Curcumin/Palygorskite Hybrid Materials
Source: Front Chem. 2021 Dec 13;9:760941. doi: 10.3389/fchem.2021.760941 (PMC8711651; doi:10.3389/fchem.2021.760941)
Supplement: Supplementary file 1 [file DataSheet1.pdf]

Supplementary Material

1 Supplementary Figures and Tables

1.1 Supplementary Figures

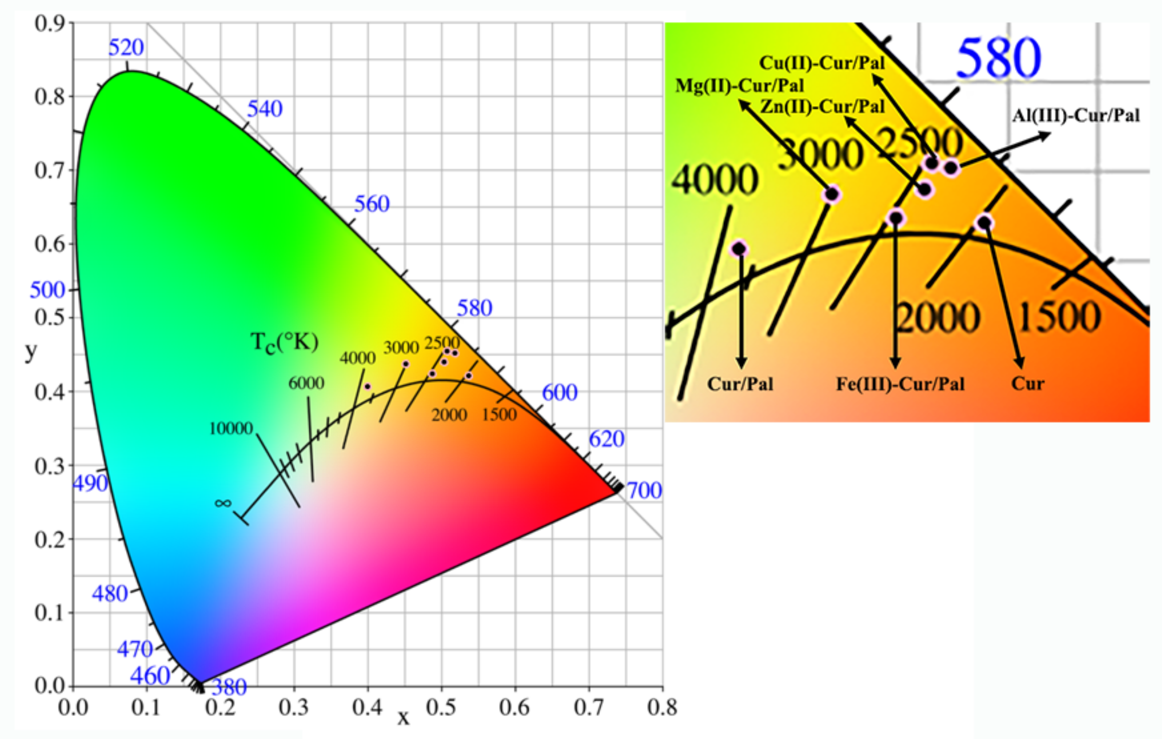

Figure S1. Chromatic CIE coordinates of Cur, Cur/Pal and metal ions-Cur/Pal hybrid materials.

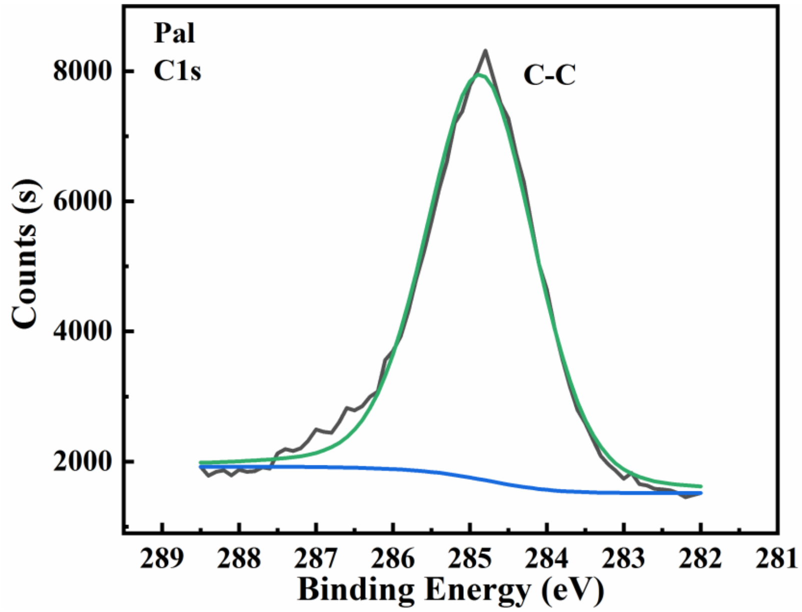

Figure S2. High-resolution XPS spectrum of C1s of Pal.

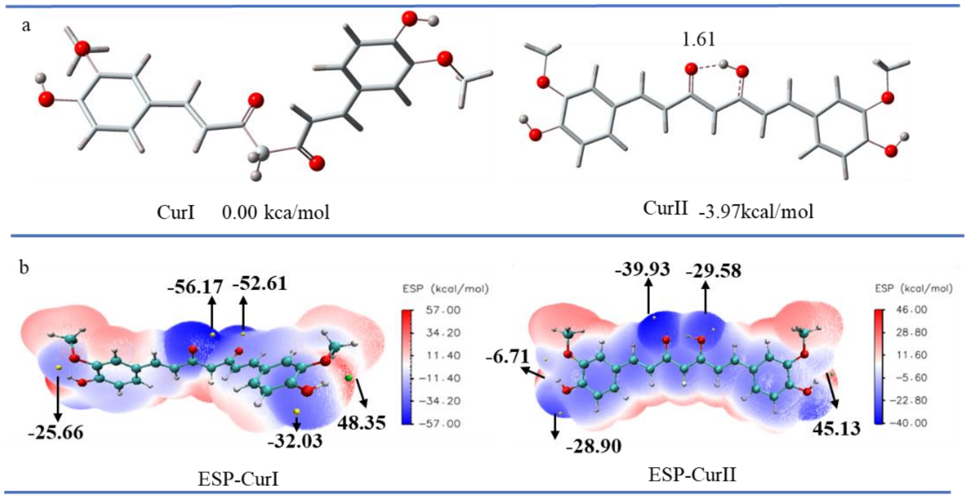

Figure S3. The optimized ketone structure and enol structure of curcumin and the Gibbs free energy at M062X/6-31G(d)/SMD level in water media; b) electrostatic potential (ESP) and the values
(kcal/mol) of the negative and positive for CurI and CurII.

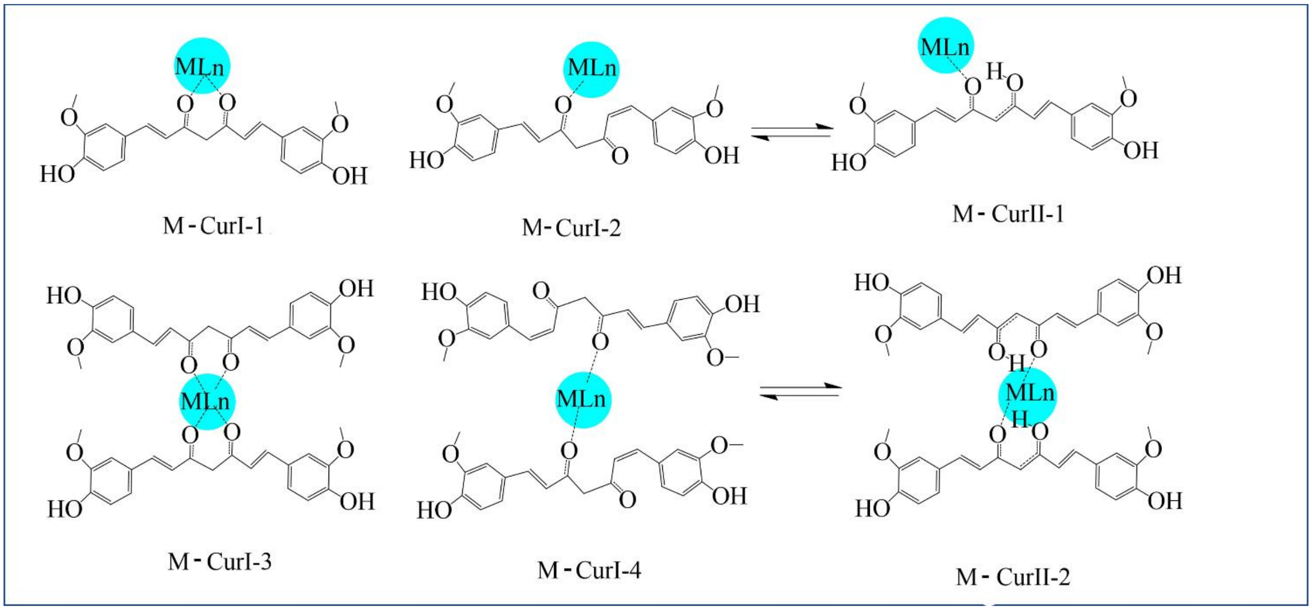

Figure S4. The possible coordination models between metal ions and Cur.

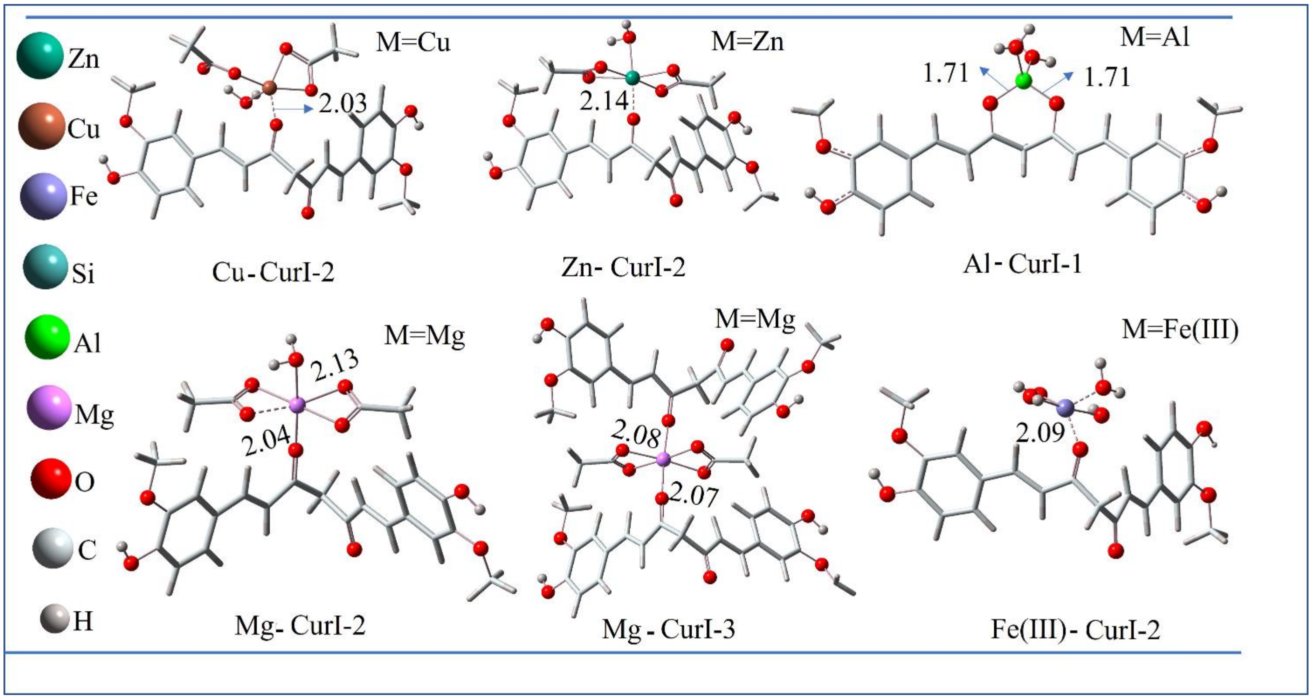

Figure S5 The optimized geometric structures of M-CurI-2, Mg-CurI-3 and Al-Cur-1 models at M06X(D3)/6-31G(d)/SDD level (distances in Å).

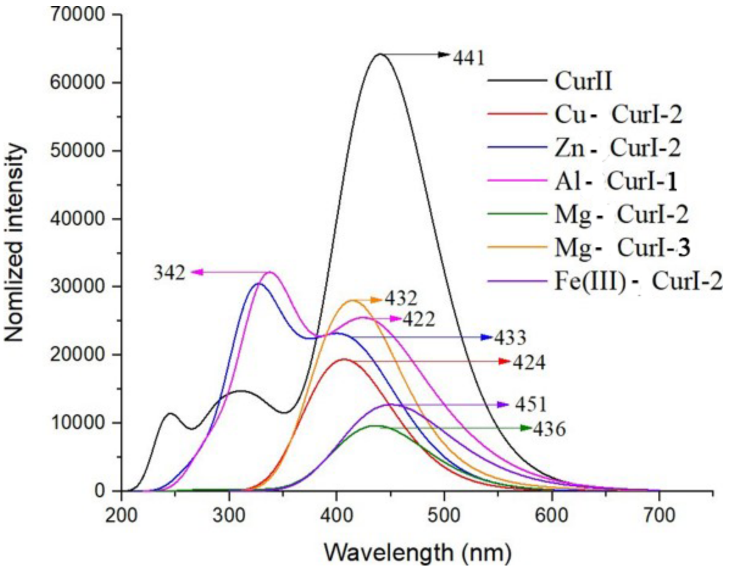

Figure S6. The calculated UV-spectra of the optimized structures (**Figure S5**) at the level of B3LYP/6-31G(d)/SMD.

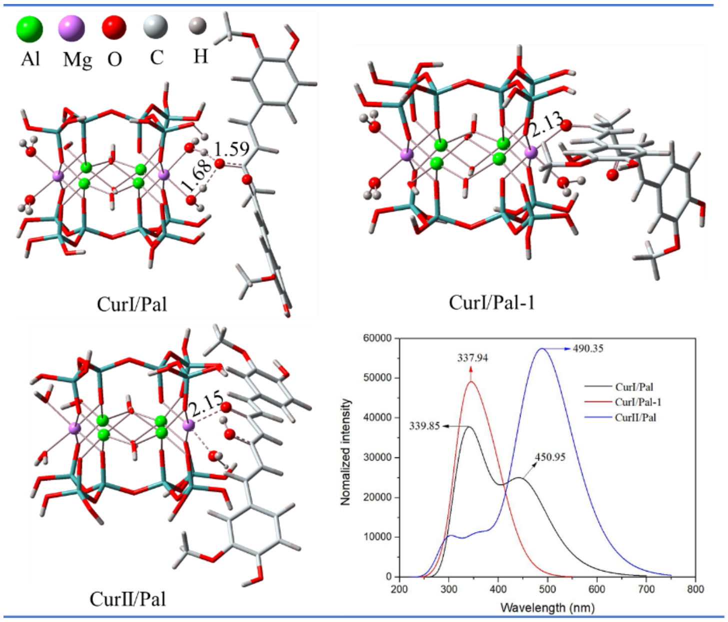

Figure S7. The possible structural models and UV spectra of Cur/Pal.

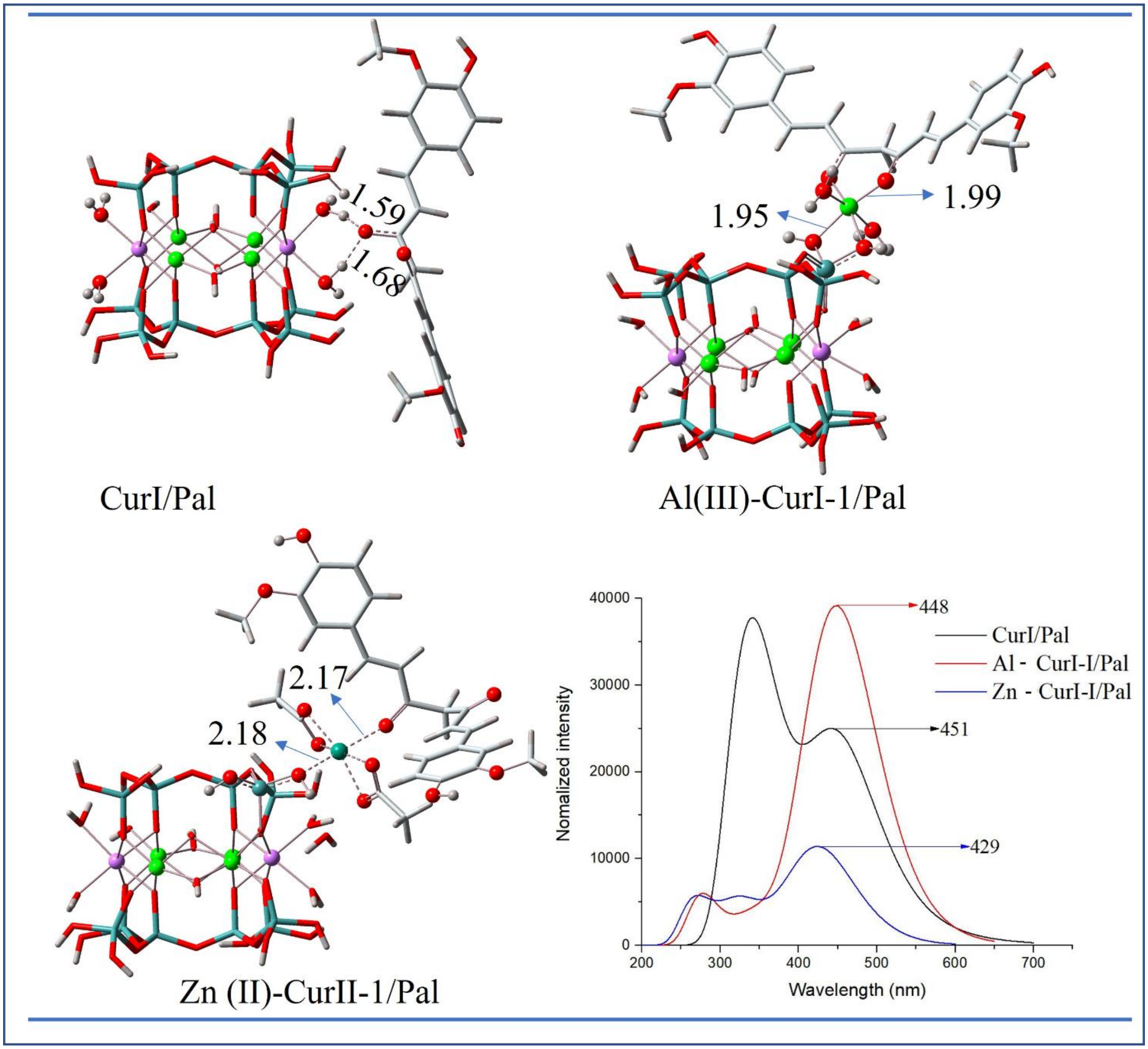

Figure S8. The optimized structures and calculated UV-spectra of Pal of CurI/Pal, Zn-CurI-1/Pal and Al-CurI-1/Pal and at M06X(D3)/6-31G(d)/SDD//B3LYP(G3BJ)/6-31G(d)/SDD (UV-spectra) level (distances in Å).

.

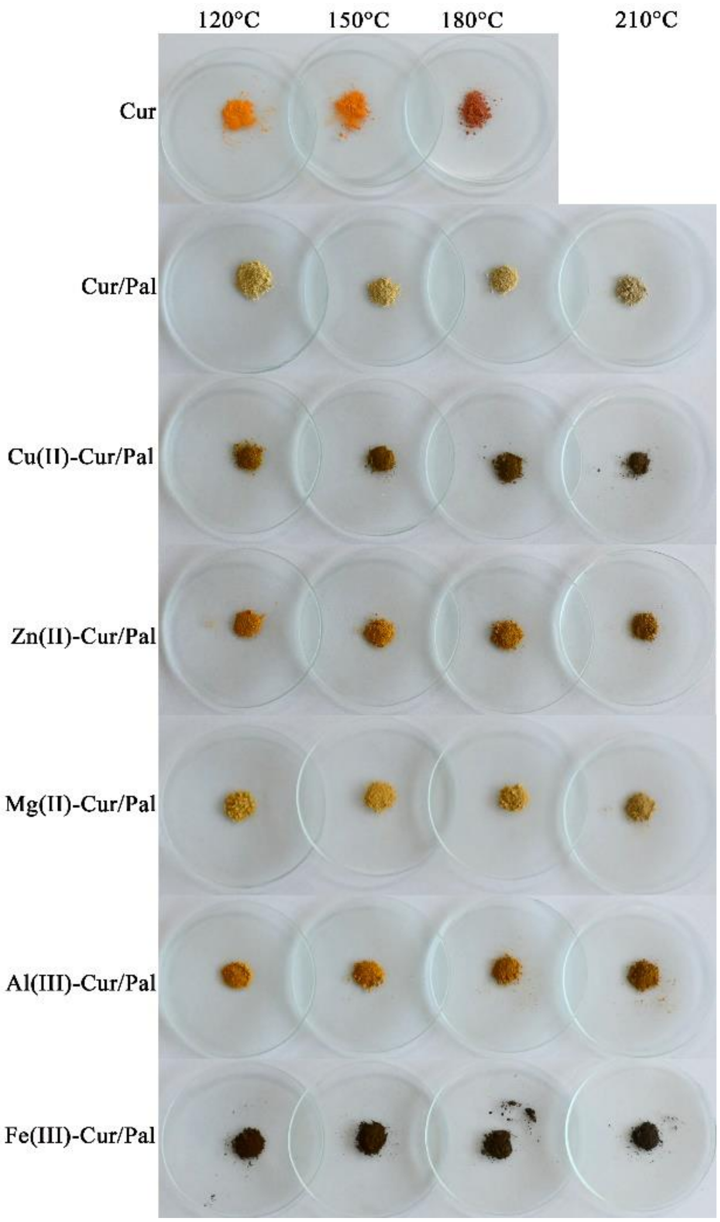

Figure S9. Digital photographs of Cur, Cur/Pal and metal ions-Cur/Pal hybrid materials after being heated at different temperatures.

1.2 Supplementary Tables

Table S1. Color parameters of Cur/Pal and metal ions-Cur/Pal hybrid materials.

Samples	Color Parameters				
	L^*	a^*	b^*	C^*	h°
Cur/Pal	68.17	4.56	34.66	34.95	82.50
Cu(II)-Cur/Pal	52.65	16.61	67.90	69.90	76.25
Zn(II)-Cur/Pal	52.07	19.14	59.46	62.47	72.16
Mg(II)-Cur/Pal	61.66	9.97	51.06	52.03	78.95
Al(III)-Cur/Pal	56.40	20.35	75.77	78.45	74.97
Fe(III)-Cur/Pal	29.11	13.00	32.63	35.13	68.27

Table S2. C% of the Cur/Pal and metal ions-Cur/Pal hybrid materials.

Samples	C%
Cur/Pal	1.26
Cu(II)-Cur/Pal	2.41
Zn(II)-Cur/Pal	2.07
Mg(II)-Cur/Pal	1.61
Al(III)-Cur/Pal	2.33
Fe(III)-Cur/Pal	3.51

Table S3. The surface composition from XPS

Samples	O1s (%)	C1s (%)	Si2p (%)	Mg1s (%)	Al2p (%)	Zn2p (%)	Fe2p (%)
Pal	49.92	18.88	20.46	4.28	5.41	-	1.06
Cur/Pal	46.26	25.63	19.2	3.05	4.69	0.26	0.91
Zn(II)-Cur/Pal	44.10	28.04	18.25	2.19	4.95	1.40	1.08
Al(III)-Cur/Pal	47.49	23.93	19.38	2.98	4.99	0.3	0.93
Fe(III)-Cur/Pal	37.31	39.68	16.24	1.69	3.83	0.24	1.01

Table S4. XPS analysis of C1s.

Sample	Element	Position (eV)	Assignment
Cur	C1s	284.66	C-H/C-C
		284.91	C-H/C-C
		286.45	C=O
Pal	C1s	284.86	C-H/C-C
Cur/Pal	C1s	284.27	C-H/C-C
		284.72	C-H/C-C
		285.22	C-O
Zn(II)-Cur/Pal	C1s	284.10	C-H/C-C
		284.72	C-H/C-C
		285.31	C-O
		286.61	C=O
Al(III)-Cur/Pal	C1s	284.31	C-H/C-C
		284.98	C-H/C-C
		285.61	C-O
Fe(III)-Cur/Pal	C 1s	283.99	C-H/C-C
		284.72	C-H/C-C
		285.24	C-O
